# Supplementary material for: Association between Prehospital Visits and Poor Health Outcomes in Korean Acute Stroke Patients: A National Health Insurance Claims Data Study
Source: Int J Environ Res Public Health. 2023 Jan 31;20(3):2488. doi: 10.3390/ijerph20032488 (PMC9915235; doi:10.3390/ijerph20032488)
Supplement: Supplementary file 1 [file ijerph-20-02488-s001.zip › ijerph-2162640-supplementary.pdf]

Table S1. Participants' characteristics of ischemic stroke group and hemorrhagic stroke group (n = 58,418).

	Ischemic stroke (n = 41,341)			Hemorrhagic stroke (n = 17,077)		
	Prehospital visit		P-value	Prehospital visit		P-value
	No	Yes		No	Yes	
N (%)	34,518 (83.5)	6,823 (16.5)	<0.001	12,908 (75.6)	4,169 (24.4)	<0.001
Age, years	68.0±13.8	67.9±13.6	0.520	61.1±15.3	60.3±14.6	<0.001
< 65	13,787 (83.6)	2,696 (16.4)	0.510	7,679 (74.5)	2,624 (25.5)	<0.001
≥ 65	20,731 (83.4)	4,127 (16.6)		5,229 (77.2)	1,545 (22.8)	
Sex			<0.001			0.009
Male	19,852 (82.6)	4,178 (17.4)		7,217 (76.7)	2,193 (23.3)	
Female	14,666 (84.7)	2,645 (15.3)		6,102 (74.7)	2,067 (25.3)	
Insurance status			0.005			<0.001
Medical aid	2,413 (85.1)	421 (14.9)		841 (80.1)	209 (19.9)	
Q1	6,108 (83.1)	1,243 (16.9)		2,444 (73.9)	864 (26.1)	
Q2	4,034 (84.4)	745 (15.6)		1,642 (74.9)	551 (25.1)	
Q3	5,105 (82.3)	1,097 (17.7)		2,057 (74.3)	711 (25.7)	
Q4	6,835 (83.3)	1,373 (16.7)		2,510 (75.4)	821 (24.6)	
Q5	10,023 (83.8)	1,944 (16.2)		3,414 (77.1)	1,013 (22.9)	
Urbanization			<0.001			<0.001
High	22,748 (87.0)	3,377 (13.0)		8,976 (79.9)	2,262 (20.1)	
Low	11,770 (77.4)	3,446 (22.6)		3,932 (67.3)	1,907 (32.7)	
Hypertension			<0.001			<0.001
Yes	17,813 (82.5)	3,791 (17.5)		5,374 (73.1)	1,973 (26.9)	
No	16,705 (84.6)	3,032 (15.4)		7,534 (77.4)	2,196 (22.6)	
Ischemic heart disease			0.007			0.008
Yes	3,888 (84.9)	692 (15.1)		958 (78.7)	259 (21.3)	
No	30,630 (83.3)	6,131 (16.7)		11,950 (75.3)	3,910 (24.7)	
Congestive heart failure			0.110			0.020
Yes	3,308 (84.4)	612 (15.6)		727 (78.8)	196 (21.2)	
No	31,210 (83.4)	6,211 (16.6)		12,181(75.4)	3,973 (24.6)	
Diabetes mellitus			0.680			0.290
Yes	2,750 (84.4)	510 (15.6)		630 (80.8)	150 (19.2)	
No	31,768 (83.4)	6,313 (16.6)		12,278 (75.3)	4,019 (24.7)	
Arrhythmia			0.003			0.891
Yes	967 (86.7)	148 (13.3)		185 (75.2)	61 (24.8)	
No	33,551 (83.4)	6,675 (16.6)		12,723 (75.6)	4,108 (24.4)	

Data were shown as N (%) or mean value ± standard deviation (SD).

Table S2. Health outcomes in ischemic and hemorrhagic stroke patients according to prehospital visits

		Extended stay in the hospital	Readmission	Mortality
Ischemic stroke	Total (%)	58.8	50.8	4.54
	Prehospital visit group (%)	60.3	53.5	4.57
	No-prehospital-visit group (%)	58.5	50.3	4.43
	Odds ratio (95% Confidence intervals)	1.01 (0.96,1.07)	1.14* (1.08,1.21)	1.04 (0.91,1.18)
Hemorrhage stroke	Total (%)	65.3	56.0	14.6
	Prehospital visit group (%)	66.6	58.2	13.7
	No-prehospital-visit group (%)	64.8	55.2	14.9
	Odds ratio (95% Confidence intervals)	1.01 (0.93,1.09)	1.12* (1.03,1.21)	0.99 (0.89,1.11)

*P<0.05

Table S3. Odds ratios (95% confidence interval) of health outcomes in stroke patients with prehospital visits according to income status and residence area

	Extended stay in the hospital		Readmission		Death	
	OR (95% CI)	P-interaction	OR (95% CI)	P-interaction	OR (95% CI)	P-interaction
**Any kinds of stroke**						
Income status		0.55		0.46		0.56
High	1.13(1.07,1.19)		1.21(1.15,1.28)		1.10(1.00,1.22)	
Low	1.01(0.94,1.08)		1.10(1.03,1.18)		1.16(1.03,1.32)	
Urbanization		<0.001		0.90		<0.001
High	1.10(1.04,1.17)		1.17(1.11,1.24)		1.10(0.98,1.22)	
Low	1.05(0.99,1.12)		1.17(1.10,1.24)		1·16(1.04,1.30)	
**Ischemic stroke**						
Income status		<0.001		<0.001		0.27
High	1.10(1.03,1.17)		1.19(1.12,1.27)		0.94(0.80,1.11)	
Low	0.94(0.86,1.03)		1.05(0.96,1.15)		1.07(0.87,1.31)	
Urbanization		0.25		0.20		0.64
High	1.07(0.99,1.15)		1.09(1.02,1.18)		0.89(0.74,1.08)	
Low	1.00(0.93,1.08)		1.19(1.11,1.29)		1.08(0.91,1.29)	
**Hemorrhagic stroke**						
Income status		0.32		0.56		0.49
High	1.04(0.95,1.15)		1.14(1.04,1.25)		0.94(0.82,1.07)	
Low	1.04(0.93,1.18)		1.09(0.97,1.23)		0.91(0.77,1.07)	
Urbanization		0.35		0.06		0.57
High	1.02(0.93,1.13)		1.20(1.09,1.32)		0.94(0.81,1.07)	
Low	1.05(0.93,1.18)		1.02(0.91,1.14)		0.92(0.79,1.08)	

Adjusting for age, sex, income, urbanization and medical history (hypertension, ischemic heart disease, congestive heart failure, diabetes mellitus and arrhythmia). Selective stratification variable (income or urbanization) was not included in the analysis.
